# Imagem Cardiovascular em Pacientes com COVID-19

**DOI:** 10.36660/abc.20200881

**Published:** 2020-11-18

**Authors:** Gabriel Blacher Grossman, Ronaldo de Souza Leão Lima

**Affiliations:** 1 Clínica Cardionuclear Porto AlegreRS Brasil Clínica Cardionuclear, Porto Alegre, RS - Brasil; 2 Hospital Moinhos de Vento Porto AlegreRS Brasil Hospital Moinhos de Vento, Porto Alegre, RS - Brasil; 3 Cardiologia Nuclear e Reabilitação Cardiovascular Departamento de Ergometria Rio de JaneiroRJ Brasil Departamento de Ergometria, Exercício, Cardiologia Nuclear e Reabilitação Cardiovascular (DERC/SBC), Rio de Janeiro, RJ - Brasil; 4 Universidade Federal do Rio de Janeiro Rio de JaneiroRJ Brasil Universidade Federal do Rio de Janeiro, Rio de Janeiro, RJ - Brasil; 5 Laboratório Diagnóstico das Américas Rio de JaneiroRJ Brazil Laboratório Diagnóstico das Américas, Rio de Janeiro, RJ - Brazil; 6 Clínica Fonte Imagem Rio de JaneiroRJ Brasil Clínica Fonte Imagem, Rio de Janeiro, RJ - Brasil; 7 Casa de Saúde São José Rio de JaneiroRJ Brasil Casa de Saúde São José, Rio de Janeiro, RJ - Brasil; 8 Sociedade Brasileira de Cardiologia Departamento de Imagem Cardiovascular Rio de JaneiroRJ Brasil Departamento de Imagem Cardiovascular - Sociedade Brasileira de Cardiologia (DIC/SBC), Rio de Janeiro, RJ - Brasil; 9 Sociedade de Cardiologia do Estado do Rio de Janeiro Rio de JaneiroRJ Brasil Sociedade de Cardiologia do Estado do Rio de Janeiro (SOCERJ), Rio de Janeiro, RJ - Brasil

**Keywords:** Coronavírus, Betacoronavírus, Pneumonia, Tomografia Computadorizada, Imagem Cardiovascular, Medicina Nuclear

**Caro Editor,**

No último número dos *Arquivos Brasileiros de Cardiologia*, Costa et al. revisou o papel da imagem cardiovascular e dos procedimentos intervencionistas em pacientes com infecção pelo novo coronavírus.^1^

Embora seja pouco provável que os procedimentos de medicina nuclear desempenhem um papel no diagnóstico primário de COVID-19 e provavelmente por este motivo não foram mencionados na revisão, a doença pode ser detectada incidentalmente em pacientes infectados mas assintomáticos que são submetidos a exames para outras indicações tendo implicações potencialmente relevantes para o manejo adicional destes pacientes. A pneumonia associada à COVID-19 é ávida por 18F-FDG e pode ser detectada como um achado incidental em pacientes assintomáticos que são submetidos à tomografia por emissão de pósitrons/tomografia computadorizada (PET/TC) para indicações oncológicas em regiões com alta prevalência de COVID-19.^2^ Da mesma maneira, achados incidentais podem ser detectados na TC usada para correção de atenuação em estudos de perfusão miocárdica. As imagens de TC adquiridas para correção de atenuação devem ser interpretadas no contexto de possíveis achados pulmonares de COVID-19.

Além disso, a captação extra-cardíaca difusa de Tc-99m sestamibi observada nos pulmões de pacientes com COVID-19 que são submetidos à cintilografia de perfusão miocárdica (CPM) pode ser explicada pelo aumento da permeabilidade vascular em relação à inflamação pulmonar, bem como pela captação celular em macrófagos ativados e fibroblastos ricos em mitocôndrias.^3^

A distinção entre pacientes de alto e baixo risco em termos de suspeita de COVID-19 serve para reduzir as chances de disseminação intra-institucional da doença, bem como para facilitar ou simplificar o rastreamento do contato. Também é imperativo considerar as indicações e a urgência da CPM durante esta pandemia. Os médicos solicitantes devem discutir e justificar a urgência do procedimento com um cardiologista ou médico nuclear se a CPM for solicitada para um paciente com COVID-19 confirmado ou suspeito, a fim de reduzir a exposição desnecessária dos profissionais de saúde a riscos de infecção.^4^ Neste cenário, é importante optar pelo protocolo de menor duração e considerar um protocolo de imagem de estresse primeiro.^5^

O teste de esforço para a CPM tem sido identificado como um procedimento de alto risco em termos de produção de gotículas. Consequentemente, o estresse farmacológico tem sido recomendado em vez do estresse por exercício em esteira, e a equipe médica e de enfermagem que atende pacientes com suspeita de infecção deve usar máscaras N95 com equipamento de proteção individual adequado.^5^

A embolia pulmonar (EP) é uma complicação importante associada à doença COVID-19, bem como um potencial diagnóstico diferencial no desconforto respiratório súbito. Em pacientes com contra-indicações para meios de contraste iodados, a tomografia por emissão de fóton único (SPECT) de perfusão pulmonar, usando albumina macroagregada marcada com [99mTc], é uma alternativa potencial. Devido ao alto risco de produção de aerossol associado à ventilação (aerossol marcado com [99mTc]), a Sociedade de Medicina Nuclear e Imagem Molecular da América do Norte, em uma primeira comunicação de Zuckier et al., desencorajou o uso da combinação clássica de imagem de ventilação-perfusão em pacientes com COVID-19,^6^ sendo esta contra-indicação flexibilizada recentemente pela mesma Sociedade. A varredura de ventilação não deve ser realizada em qualquer paciente com infecção por COVID-19 conhecida ou suspeita; consequentemente, tem sido proposto um algoritmo baseado em radiografia de tórax e exames de SPECT apenas de perfusão em pacientes sem opacidades pulmonares. Isto exclui todos os pacientes com infiltrados pulmonares e, portanto, a maioria dos pacientes com doença crítica associada à COVID-19.

Em resumo, a medicina nuclear é fundamental para o manejo de doenças cardiovasculares.^7^^–^^11^ na cardiologia clínica de rotina, mas não é a primeira linha de abordagem para pacientes com COVID-19. Apesar disso, seus procedimentos podem eventualmente ajudar no manejo desses pacientes. Além disso, os exames de perfusão pulmonar podem ser uma alternativa quando há suspeita de EP. É importante ressaltar que cardiologistas nucleares e médicos nucleares devem estar cientes dos achados incidentais em pacientes assintomáticos com COVID-19 e devem otimizar os protocolos de CPM, quando o procedimento for necessário.
