# Supplementary material for: Imagem Cardiovascular em Pacientes com COVID-19
Source: Arq Bras Cardiol. 2020 Nov 18;115(5):973–4. [Article in Portuguese] doi: 10.36660/abc.20200881 (PMC8452199; doi:10.36660/abc.20200881)
Supplement: Translation

# Translation: Cardiovascular Imaging in Patients with COVID-19

**Authors:** Gabriel Blacher Grossman, Ronaldo de Souza Leão Lima

**Affiliations:** 1 Porto AlegreRS Brazil Clínica Cardionuclear, Porto Alegre, RS - Brazil; 2 Porto AlegreRS Brazil Hospital Moinhos de Vento, Porto Alegre, RS - Brazil; 3 Rio de JaneiroRJ Brazil Departamento de Ergometria, Exercício, Cardiologia Nuclear e Reabilitação Cardiovascular (DERC/SBC), Rio de Janeiro, RJ - Brazil; 4 Rio de JaneiroRJ Brazil Universidade Federal do Rio de Janeiro, Rio de Janeiro, RJ - Brazil; 5 Rio de JaneiroRJ Brasil Laboratório Diagnóstico das Américas, Rio de Janeiro, RJ - Brasil; 6 Rio de JaneiroRJ Brasil Clínica Fonte Imagem, Rio de Janeiro, RJ - Brasil; 7 Rio de JaneiroRJ Brazil Casa de Saúde São José, Rio de Janeiro, RJ - Brazil; 8 Rio de JaneiroRJ Brazil Departamento de Imagem Cardiovascular - Sociedade Brasileira de Cardiologia (DIC/SBC), Rio de Janeiro, RJ - Brazil; 9 Rio de JaneiroRJ Brazil Sociedade de Cardiologia do Estado do Rio de Janeiro (SOCERJ), Rio de Janeiro, RJ - Brazil

**Keywords:** Coronavirus, Betacoronavirus, Pneumonia, Computed Tomography, Image Cardiovascular, Nuclear Medicine

**Dear Editor,**

In the latest issue of the *Arquivos Brasileiros de Cardiologia*, Costa et al. reviewed the role of cardiovascular imaging and interventional procedures in patients with novel coronavirus infection.^1^

Although nuclear medicine procedures are unlikely to play a role in the primary diagnosis of COVID-19, which is probably the reason that they were not mentioned in this review, the disease may be detected incidentally in asymptomatic infected patients who are undergoing scans for other indications, and it has potentially relevant implications for further management. COVID-19-associated pneumonia is 18F-FDG-avid, and it may be detected as an incidental finding in asymptomatic patients who are undergoing positron emission tomography/computed tomography (PET/CT) for oncologic indications in regions with a high COVID-19 prevalence.^2^ Similarly, incidental findings may be detected in CT used for attenuation correction in myocardial perfusion studies. CT images acquired for attenuation correction should be interpreted in the context of possible COVID-19 pulmonary findings.

Also, the diffuse extra-cardiac Tc-99m sestamibi signal observed in the lungs of COVID-19 patients who are submitted to myocardial perfusion imaging (MPI) scan might be explained by increased vascular permeability in relation to lung inflammation, as well as by the cellular uptake in activated macrophages and fibroblasts rich in mitochondria.^3^

Distinguishing high-risk and low-risk patients in terms of suspicion of COVID-19 serves to reduce the chance of intra-institutional spread of the disease, as well as to facilitate or simplify contact tracing. It is also imperative to consider the indications and urgency of MPI during this pandemic. Referring physicians should discuss and justify the urgency of the procedure with a nuclear cardiologist or physician if an MPI is ordered for patients with confirmed or suspected COVID-19, in order to reduce unnecessary exposure of healthcare workers to risks of infection.^4^ In this scenario, it is important to select the protocol with the shortest duration and to consider a stress first imaging protocol.^5^

Exercise stress testing for MPI has been identified as a high-risk procedure in terms of droplet production. Consequently, pharmacological stress has been recommended over treadmill exercise stress, and medical and nursing staff who attend patients with suspected infection are required to wear N95 masks with appropriate personal protective equipment.^5^

Pulmonary embolism (PE) is an important complication associated with COVID-19 disease, as well as a potential differential diagnosis in sudden respiratory distress. In patients with contraindications for iodinated contrast media, perfusion single-photon emission tomography (SPECT) using [99mTc]-labeled macroaggregated albumin is a potential alternative. Due to the high risk of aerosol production associated with ventilation ([99mTc]-labeled aerosol) scans, the North American Society of Nuclear Medicine and Molecular Imaging, in a recent communication by Zuckier et al., has discouraged use of the classic imaging combination of ventilation-perfusion in patients with COVID-19,^6^ this contraindication being recently relaxed by the same Society. Ventilation scans should be omitted in any patient with known or suspected COVID-19 infection; a chest X-ray based algorithm has consequently been proposed, with perfusion only SPECT scans in patients without pulmonary opacities. This excludes all patients with pulmonary infiltrates and, therefore, the majority of patients with critical illness associated with COVID-19.

In summary, nuclear medicine is pivotal for managing cardiovascular disease^7^^–^^11^ in routine clinical cardiology, but it is not the first line approach for patients with COVID-19. Nevertheless, these procedures may eventually help in the management of these patients. Moreover, lung perfusion scans can be an alternative when PE is suspected. Importantly, nuclear cardiologists and nuclear medicine physicians must be aware of incidental findings in asymptomatic patients with COVID-19, and they should optimize MPI protocols, when the procedure is necessary.
